# Adolescents’ exposure to tobacco and alcohol content in YouTube music videos

**DOI:** 10.1111/add.12835

**Published:** 2015-01-28

**Authors:** Jo Cranwell, Rachael Murray, Sarah Lewis, Jo Leonardi‐Bee, Martin Dockrell, John Britton

**Affiliations:** ^1^ UK Centre for Tobacco and Alcohol Studies, Division of Epidemiology and Public HealthUniversity of NottinghamClinical Sciences Building, City HospitalNottinghamUK; ^2^ Public Health EnglandLondonUK

**Keywords:** Adolescent exposure, alcohol, music videos, new media, tobacco, YouTube

## Abstract

**Aims:**

To quantify tobacco and alcohol content, including branding, in popular contemporary YouTube music videos; and measure adolescent exposure to such content.

**Design:**

Ten‐second interval content analysis of alcohol, tobacco or electronic cigarette imagery in all UK Top 40 YouTube music videos during a 12‐week period in 2013/14; on‐line national survey of adolescent viewing of the 32 most popular high‐content videos.

**Setting:**

Great Britain.

**Participants:**

A total of 2068 adolescents aged 11–18 years who completed an on‐line survey.

**Measurements:**

Occurrence of alcohol, tobacco and electronic cigarette use, implied use, paraphernalia or branding in music videos and proportions and estimated numbers of adolescents who had watched sampled videos.

**Findings:**

Alcohol imagery appeared in 45% [95% confidence interval (CI) = 33–51%] of all videos, tobacco in 22% (95% CI = 13–27%) and electronic cigarettes in 2% (95% CI = 0–4%). Alcohol branding appeared in 7% (95% CI = 2–11%) of videos, tobacco branding in 4% (95% CI = 0–7%) and electronic cigarettes in 1% (95% CI = 0–3%). The most frequently observed alcohol, tobacco and electronic cigarette brands were, respectively, Absolut Tune, Marlboro and E‐Lites. At least one of the 32 most popular music videos containing alcohol or tobacco content had been seen by 81% (95% CI = 79%, 83%) of adolescents surveyed, and of these 87% (95% CI = 85%, 89%) had re‐watched at least one video. The average number of videos seen was 7.1 (95% CI = 6.8, 7.4). Girls were more likely to watch and also re‐watch the videos than boys, *P* < 0.001.

**Conclusions:**

Popular YouTube music videos watched by a large number of British adolescents, particularly girls, include significant tobacco and alcohol content, including branding.

## Introduction

Harmful consumption of alcohol causes a global total of 2.5 million deaths 1, and tobacco smoking 6 million deaths 2, each year. In the United Kingdom alcohol and tobacco consumption cause, respectively, 7000 3 and 100 000 deaths 4, and together cost the UK National Health Service (NHS) at least £5.4 billion 3, 5, 6 annually. Preventing this morbidity and mortality is a clear public health priority. Because most smokers start smoking during adolescence 7, 8, and initiating alcohol consumption at a young age is a strong risk factor for dependence in later life 9, it is crucial to identify avoidable risk factors for tobacco or alcohol use in this period of development.

There is now strong evidence that adolescent exposure to paid‐for advertising and other alcohol or tobacco media imagery in the media increase subsequent alcohol 10, 11, 12, 13, 14, 15 and tobacco use 16, 17, 18, 19, 20, 21. Media exposure includes films and television programmes, in which both tobacco 22, 23, 24, 25, 26 and alcohol 13, 26, 27, 28, 29, 30, 31 imagery are common. Further, social media have provided tobacco companies with new opportunities to promote their products 32 and generate favourable attitudes towards tobacco, including intention to smoke, in young non‐smokers 33. In the 1990s and early 2000s televised music videos included significant alcohol and tobacco content 34, 35, 36. However, music videos are now viewed predominantly via on‐line channels such as YouTube, and are another potentially important source of exposure.

YouTube dominates the music video‐sharing market in the United Kingdom, was the fourth most popular UK website in 2011 37, and is particularly popular among 12–17‐year‐olds 37. In 2014 the site attracted more than 1 billion unique users 38, approximately five times more than in 2006 39. In April 2011 music videos accounted for 30% of the top 10 most viewed channels 37, and in 2007 YouTube videos were reported qualitatively to contain extensive and easily accessible tobacco imagery 40. To quantify contemporary exposure we have therefore analysed alcohol and tobacco content in YouTube music videos of the 110 most popular songs in UK music charts during the 12‐week period from 3 November to 19 January 2014. We have also used a British (GB) nationally representative on‐line survey administered to 11–18‐year‐olds between 21 March and 1 April 2014 to measure reported viewing of 32 of the most popular videos containing either tobacco or alcohol imagery.

## Methods

### Music video content

#### Procedure

We searched *YouTube* for videos of all records listed in the *Official Singles Chart UK Top 40*, which is used by BBC Radio 1, BBC Radio 2, MTV and Music Week, and is a joint trade association venture between the British Music Industry (BMI) and the Entertainment Retailers Association (ERA) 41; or the *Vodafone Big Top 40* music chart generated from *iTunes* sales and aired weekly by most major UK commercial radio stations 42, in the 12 weeks ending Sunday 3 November 2013 to Sunday 19 January 2014. Of the 130 different songs listed in the two charts during the study period, YouTube videos were available for 110 (see Supporting information, Appendix S1). Of these, 76 (69%) were uploaded to YouTube by VEVO, a digital music platform that syndicates to multiple global on‐line sites; 28 (26%) directly by the artist or their record company, four (4%) by a film or TV company and two (2%) from sources such as a YouTube music group.

#### Data analysis

Videos were analysed using a method adapted from that described previously for television and film content 22, 27 to provide semi‐quantitative estimates of alcohol, tobacco and electronic cigarette (or other nicotine‐containing device) content. We analysed visual and lyrical content of videos using 10‐second intervals, coding each for the presence or absence of alcohol, tobacco or electronic cigarettes in the following categories:

*Alcohol use*: actual consumption of an alcoholic drink by any character.
*Implied alcohol use*: open bottles of or glasses appearing to hold alcoholic drinks, drunken behaviour or other appearance implying alcohol consumption but without actual use.
*Alcohol paraphernalia*: bottles, glasses or other materials associated with alcohol (for example, a shot of a bar containing alcohol bottles and glasses) without actual or implied use.
*Alcohol brand appearance*: clear and unambiguous alcohol branding on a product consumed or otherwise visible in the scene, or in advertisements, logos or other recognizable branded material.
*Any alcohol content*: occurrence of any of the above.
*Tobacco use*: any use of a tobacco product by any character, coded as cigarette, cigar, pipe or other (such as water pipe or chewing tobacco, inhaling/exhaling smoke).
*Implied tobacco use*: a smoky atmosphere, a character holding a cigarette but not seen smoking it, or any other implied but not actual tobacco use.
*Tobacco paraphernalia*: tobacco or tobacco‐related materials, such as a cigarette or other tobacco pack, matches, lighter, ashtray, no smoking or smoking area signs, but without actual or implied use.
*Tobacco brand appearance*: clear and unambiguous tobacco branding, including cigarette or other tobacco packs, and branded merchandising.
*Any tobacco content*: the occurrence of any of the above.
*Electronic cigarette content*: coded as for tobacco use, implied use or brand appearance.

All coding was carried out independently by authors J.C. and R.M., and any differences resolved by discussion. Repeated appearances in the same category during any single 10‐second interval were coded as a single event, and appearances in different categories as separate events, with the exception of brands, for which different brands were counted as separate events. If the same appearance, regardless of category, crossed into the next 10‐second interval it was coded as a new single event. Where different categories of appearance of alcohol, tobacco or electronic cigarettes occurred simultaneously (for example, actual and implied use of alcohol) the episode was coded under the higher of the categories as ranked above. Any partial intervals at the end of a video were counted as a full 10‐second interval. Content data were recorded in a Microsoft Excel 2010 spreadsheet and analysed using basic descriptive statistics procedures in the Statistical Package for the Social Sciences (IBM SPSS version 22).

### Music video survey

#### Procedure

We included questions on viewing the 32 most popular (highest chart position) songs with tobacco or alcohol content in a national survey of adolescents carried out by YouGov PLC and the public health charity Action on Smoking and Health (ASH). In accordance with YouGov practice, people aged 16–18 years were recruited by direct e‐mail invitations to a random sample of panellists from a database of individuals who had consented to be contacted, informing them of the survey and inviting them to take part. Adolescents aged 11–15 years were recruited by e‐mailing parents or legal guardians from the YouGov database and asking them, after reading the study information, to explain the nature of the study and what was required, and request oral assent. Consenting respondents then followed a URL link to complete the on‐line survey, and data from the two samples were merged and weighted to be representative of age, gender and region (11 GB government regions in total) in relation to Office for National Statistics census data. The 32 music videos were presented in randomized order for each participant within the following questions: ‘Which, if any, of the following music videos have you seen?’ and ‘Which, if any, of the following music videos have you seen more than once?’. Respondents were asked to tick all that applied. The YouGov survey was carried out in accordance with both the MRS (Market Research Society) and the BPC (British Polling Council) codes of practice.

#### Data analysis

Survey data were analysed using basic descriptive statistics procedures and 95% confidence intervals (CI) were estimated in Microsoft Excel 2010.

## Results

### Content analysis

The mean duration of videos was 241 seconds (range = 158–433 seconds), comprising a total of 26 466 seconds of screen time and 2711 10‐second intervals for analysis.

There were alcohol appearances 376 intervals (14% of all coding intervals) in 49 (45%) videos, and tobacco appearances in 83 intervals (3%) in 24 (22%) videos. Electronic cigarettes appeared in 21 intervals in two videos. When categorized by genre 43, the highest proportion of videos containing alcohol or tobacco were pop, hip‐hop/rap, dance and alternative (Table 1). Overall, 83% of videos containing tobacco also included alcohol and 41% of videos containing alcohol also included tobacco.

**Table 1:** Alcohol, tobacco or electronic cigarette content of videos by genre.

			*Alcohol*			*Tobacco*			*Electronic cigarette*		
*Video genre*	*Overall frequency (%)*		*Frequency (%)*		*Number of intervals (any alcohol)*	*Frequency (%)*		*Number of intervals (any tobacco)*	*Frequency (%)*		*Number of intervals (any electronic)*
Pop	56	(51%)	22	(20%)	225	8	(7%)	24	1	(1%)	6
Electronic	4	(4%)	2	(2%)	9	3	(3%)	19	0	(0%)	0
Hip‐hop/Rap	12	(11%)	7	(6%)	58	4	(4%)	8	1	(1%)	15
Dance	11	(10%)	7	(6%)	24	1	(1%)	1	0	(0%)	0
Alternative	7	(6%)	5	(5%)	27	5	(5%)	27	0	(0%)	0
House	4	(4%)	2	(2%)	18	1	(1%)	2	0	(0%)	0
R&B/Soul	1	(1%)	1	(1%)	4	1	(1%)	1	0	(0%)	0
Soundtrack	3	(3%)	1	(1%)	1	0	(0%)	0	0	(0%)	0
Charity	2	(2%)	0	(0%)	0	0	(0%)	0	0	(0%)	0
Rock	4	(4%)	1	(1%)	1	1	(1%)	1	0	(0%)	0
Singer/ songwriter	2	(2%)	0	(0%)	0	0	(0%)	0	0	(0%)	0
Country	1	(1%)	0	(0%)	0	0	(0%)	0	0	(0%)	0
Total	110	(100%)	49	(45%)	376	24	(22%)	83	2	(2%)	21
				(95 % CI = 33–51%)			(95% CI = 13–27%)			(95% CI = 0–4%)	

CI = confidence interval.

#### Alcohol

**Figure 1:**
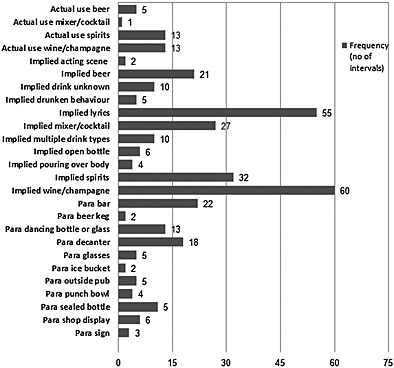
Detail of alcohol content in videos (para = paraphernalia, implied = implied use)

Episodes of actual alcohol use occurred in 32 intervals in 17 (16%) videos, and implied use in 232 intervals in 13 (12%) videos. Verbal references, mainly in lyrics, comprised 25% of all implied use. Episodes of alcohol paraphernalia occurred in 91 intervals in 20 (18%) videos and alcohol branding in 23 intervals in eight (7%) videos (Fig. 1).

Videos containing alcohol brands comprised ‘Drunk in Love’ by Beyoncé (featuring Jay Z) which mentioned in the lyrics both the cognac brand D'USSE™: ‘That D'USSÉ is the sh*t if I do say so myself’ and the champagne brand Armand de Brignac™: ‘Boy, I'm drinking, get my brain right, Armand de Brignac, gangster wife’. Jay Z is the ‘spokesperson’ for the D'USSE™ brand 44, 45. Voli™ vodka featured both in the lyrics and on screen in ‘Timber’ by Pitbull (featuring K$sha). Pitbull is listed on‐line as the ‘shareholder’, ‘official spokesman and ambassador’ of the Voli vodka brand 46. The Grey Goose™ vodka brand was mentioned in the lyrics of ‘Royals’ by Lorde: ‘…Grey Goose, trippin’ in the bathroom’. Hennessy™ cognac was observed on screen in the video ‘Thank you’ by Busta Rhymes (featuring Q‐Tip, Kanye West and Lil Wayne), as was Cavoda™ vodka in ‘Show me’ by Kid Ink (ft. Chris Brown). The video ‘Blurred lines’ by Robin Thicke (featuring T.I. and Pharrell Williams) highlighted the Remy Martin™ cognac brand on screen. The most frequently observed alcohol brand was Absolut Tune™, which featured in four coding intervals in ‘All Night’ by Icona Pop, who are the ‘face’ of the Absolut Tune brand 47.

#### Tobacco

**Figure 2:**
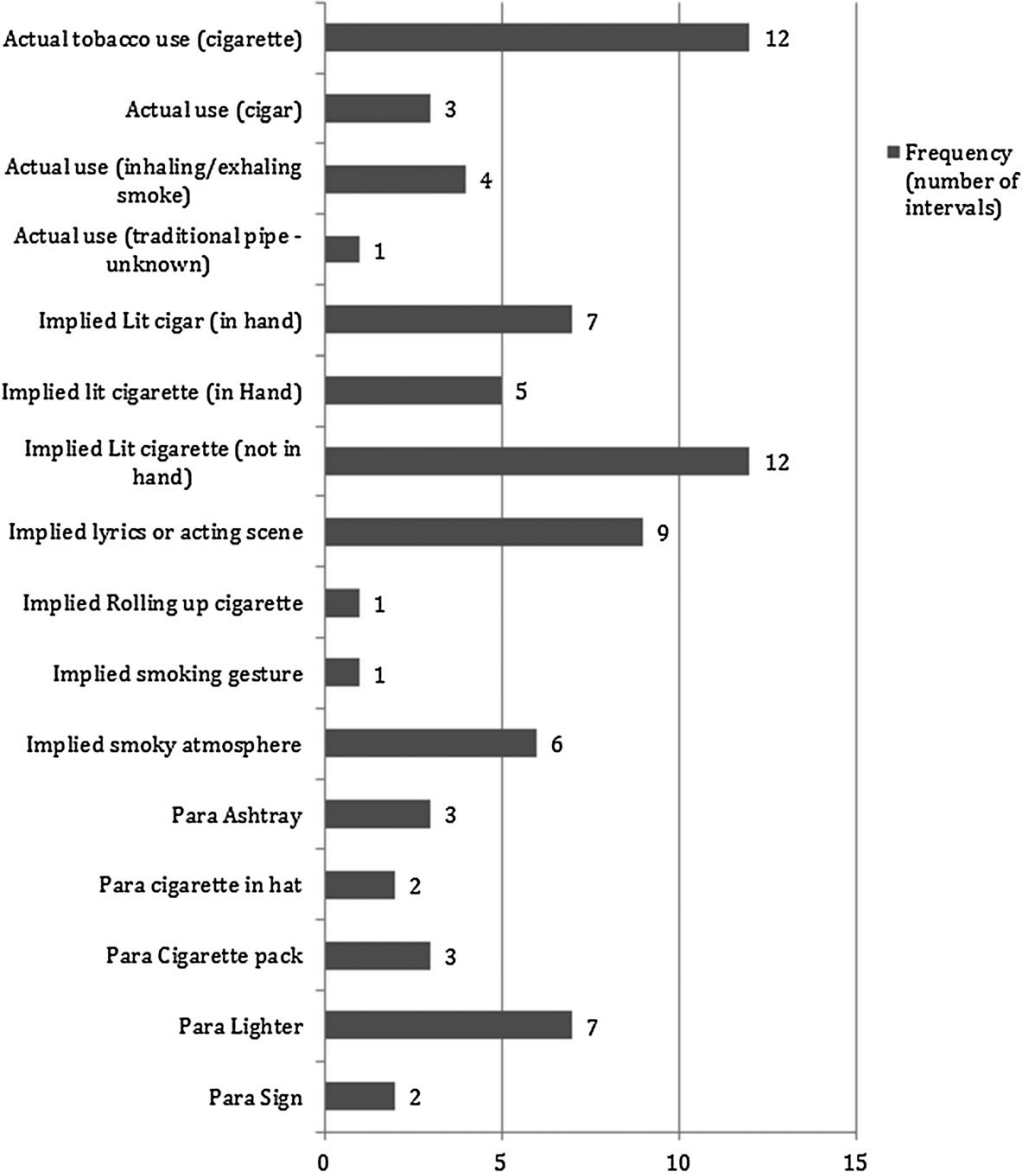
Detail of tobacco content of videos (para = paraphernalia, implied = implied use)

Episodes of actual tobacco use occurred in 20 intervals in 10 (9%) videos, and of implied use in 41 intervals in 10 (9%) videos. Verbal references accounted for 23% of all implied use. Tobacco paraphernalia occurred in 17 intervals in 10 (9%) videos and tobacco branding in five intervals in four (4%) videos. Details of the content are given in Fig. 2.

The most frequently observed tobacco brand was Marlboro™, which featured in three separate episodes over two videos; once in a large sign on the side of a shop in ‘Alive’ by Chase and Status, and twice in ‘Of The Night’ by Bastille, where clear branding shots of multiple Marlboro cigarette packs were featured behind a shop display counter. Camel™ cigarette branding featured in one interval in the video ‘Riptide’ by Vance Joy, and Embassy™ very briefly in ‘Love Me Again’ by John Newman.

#### Electronic cigarettes

Electronic cigarette use occurred in nine intervals in two (2%) videos, and implied use (all visual) in 10 intervals in two (2%) videos. There were no episodes of electronic cigarette paraphernalia. Electronic cigarette branding occurred in two intervals in one (1%) video, ‘Hard Out There’ by Lily Allen, in which packs of the E‐Lites™ brand were held by dancers while also using them.

### Survey of music video viewing

**Figure 3:**
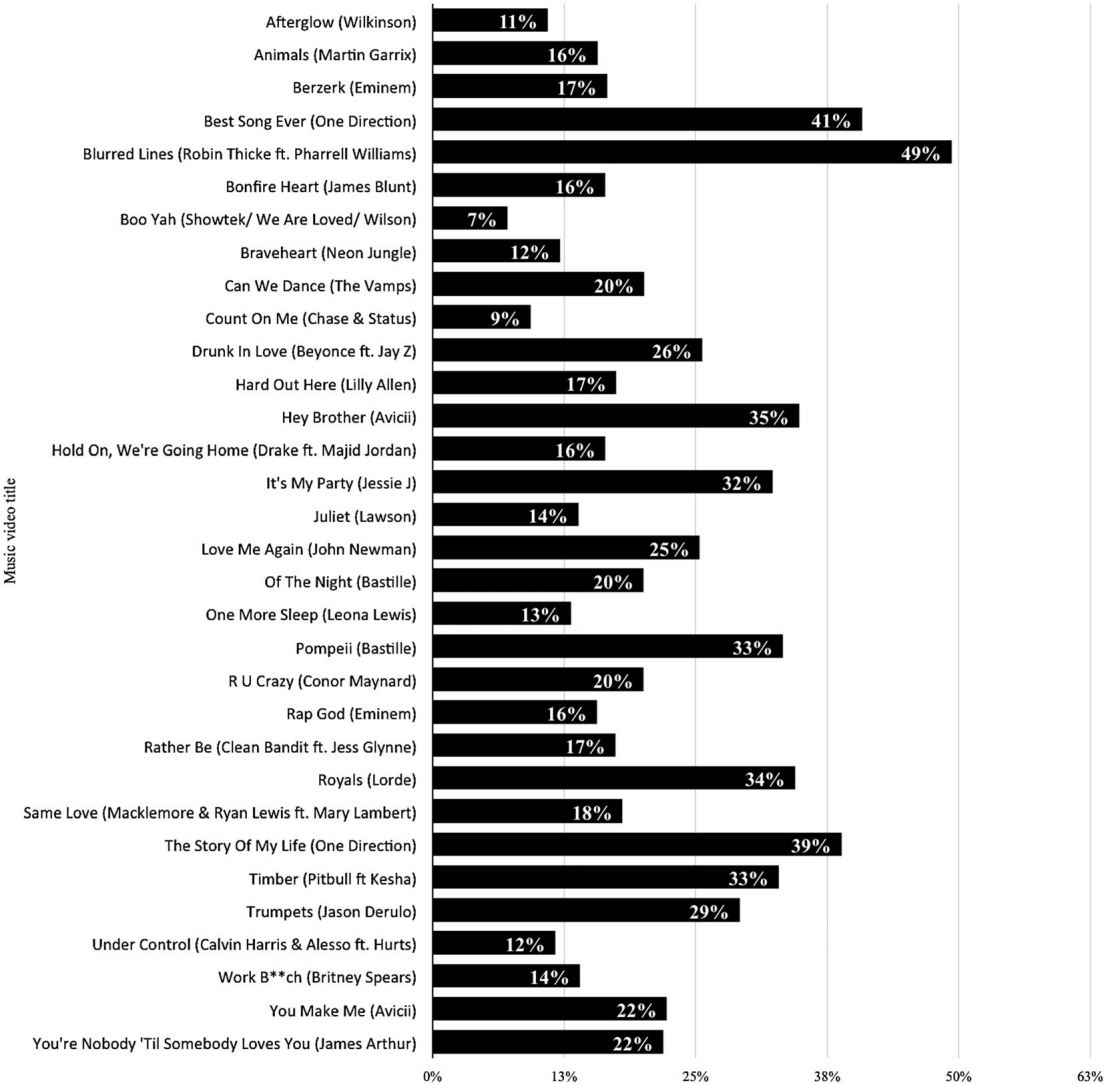
Proportion of adolescents aged 11–18 years who had watched the 32 most popular music videos

A total of 11 622 eligible participants aged 11–18 years were invited to participate in the survey and 2068 adolescents (1064 boys and 1005 girls), of whom 65% were aged less than 15 years, completed it (mean age = 14 years 7 months). Table 2 provides the main video viewing results by age and gender. Further, 529 (26%, 95% CI = 24–27%) and 247 (12%, 95% CI = 11–13%) respondents watched more than 10 and more than half of all videos, respectively. There was a significant association between gender and music video watching, suggesting that girls were more likely to watch and also re‐watch the videos than boys, χ^2^_(1, *n* = 2068)_ = 108.74, *P* < 0.001 and χ^2^_(1, *n* = 1675)_ = 12.27, *P* < 0.001, respectively. Figure 3 shows the proportion of adolescents aged 11–18 years who had watched the 32 most popular music videos. ‘Blurred Lines’ was the most watched and re‐watched video at 49% (95% CI = 47–51%) and 43% (95% CI = 40–45%), respectively.

**Table 2:** Music video viewing by age and gender.

*Age and gender*	*Mean videos watched (any video) and 95% CI*	*Viewing figures— watched any*		*Mean videos re‐watched and 95% CI*	*Viewing figures—re‐watched any*	
		*n1*	*%*		*n2*	*% n1 and 95% CI*
Boys 11–15 years *n* = 647	5.7 (5.2, 6.2)	468	72% (69%, 76%)	4.7 (4.2,5.2)	396	85% (81%, 88%)
Boys 16–18 years *n* = 416	4.6 (4.1, 5.2)	300	72% (68%, 76%)	3.6 (3.1, 4.1)	247	82% (78%, 87%)
Girls 11–15 years *n* = 614	8.8 (8.2, 9.4)	549	89% (87%, 92%)	6.1 (5.6, 6.7)	502	91% 89%, 94%)
Girls 16–18 years *n* = 391	9.2 (8.5, 10)	358	92% (89%, 94%)	6.1 (5.5, 6.7)	310	87% (83%, 90%)
Total	7.1 (6.8, 7.4)	1675	81% (79%, 83%)	5.3 (5, 5.6)	1455	87% (85%, 89%)

## Discussion

This study demonstrates that both tobacco and alcohol imagery, including branding, occur frequently in the lyrical and visual content of popular music videos and are seen by a very high proportion of young people, particularly girls. Although we were unable to measure any effect of exposure on use of tobacco or alcohol in our study, there is strong evidence that exposure to such imagery in other media increases tobacco and alcohol use. Given the high levels of exposure to YouTube content, it is therefore highly likely that exposure to this imagery has an important effect on smoking and alcohol consumption among young people.

To our knowledge, this is the first study ever to quantify music video content and measure viewing among the adolescent audience. Although our on‐line survey response rate was low, it was not atypical of web‐based survey responses, which are normally approximately 10% fewer than mail or telephone surveys 48. The respondent sample was self‐selected, but the media topic was not made salient to them before starting the survey, as the questions formed only a small component of a wider survey. As there are no established methods of coding music video content, we adapted an interval coding procedure used successfully in film and television content analysis 22, 27 to analyse the these short‐format videos. Interval coding has been criticized when used in other settings for producing more systemic errors than alternative time sampling methods, such as momentary sampling. It is, however, a more sensitive method of measurement 49. Further, because the direction of error points towards underestimation of change in high‐rate short‐duration behaviours, such as the ones typically observed in short‐burst quick‐scene changes in music videos, we aimed to reduce this by using a very short 10‐second interval duration. Overall, therefore, while underestimation may still persist in this study, the short interval duration reduces the potential for overestimation of behavioural rates, which may be problematic in longer‐duration coding intervals and which may produce less credible data.

The 2002 Tobacco Advertising and Promotion Act prohibits tobacco advertising in the United Kingdom, and defines an advertisement as anything ‘whose purpose is to promote a tobacco product, or whose effect is to do so’ 50. As exposure to tobacco imagery in the media promotes smoking, the Marlboro, Camel and Embassy imagery identified in these videos constitute advertisements, and in our view are in breach of the Act. However, while all tobacco branding was presented passively in the videos, without apparent direct endorsement by the artists involved, alcohol brands were endorsed widely and actively. Many of the videos analysed were, in effect, advertisements for specific alcohol brands which were either used or mentioned in lyrics by artists with apparent financial associations with these products. Therefore, while both the tobacco and alcohol content are likely to be significant drivers of both tobacco and alcohol consumption among young people, the alcohol content is apparently intended as such. Our results also identify the nascent use of electronic cigarette imagery in music videos, which has not, to our knowledge, been reported previously.

Earlier work on music video content has reported on the presence or absence of alcohol or tobacco imagery in complete videos broadcast on television, and found that alcohol was present in 34.5% and actual consumption in 10%, and tobacco in 10% and actual tobacco use in 8%, of a sample of televised music videos broadcast during 6 weeks in 2001 35. In our study the level of actual use of tobacco was similar (9%), but tobacco content was more than twice as prevalent (22%), while both alcohol use and content were higher. We also found slightly higher rates of tobacco brand appearances than in the earlier study, at 4% versus 1.5% for tobacco 35. In our study, mention of alcohol content in the lyrics was fairly consistent with that reported by Roberts *et al.* 28 in 1999 (23 versus 17%), with both Remy Martin and Hennessy brands featuring prominently in the present and earlier studies 28. In contrast, however, mention of tobacco was not reported by Roberts *et al*., as its presence was deemed too low. Our figures for tobacco branding and alcohol content indicate that these were also more prevalent in our sample than in an analysis of television music videos shown on four American television networks in 1994 by DuRant *et al.* 36. Thus, it appears that the prevalence of both tobacco and alcohol content is higher in contemporary digital on‐line music videos than in equivalent media from earlier years. Product placement through commercial agreements between alcohol companies and individual artists is likely to explain at least some of the increase in alcohol content. Paid tobacco product placement is now illegal in the United Kingdom, so it is not clear whether the increase in tobacco content is commercially driven.

Since 1985, most record companies have agreed voluntarily to place parental advisory labels, for example on CD packaging, on music content that is either sexually or violently explicit or involves drug use 51. Unlike TV and film, music videos are not classified according to age suitability and are not required to provide viewer advice on content relating to addictive substances such as tobacco or alcohol. Our finding that YouTube music videos include significant tobacco and alcohol content and that, in relation to alcohol, this is often overt brand endorsement or promotion, suggests that this policy should change. The use of celebrity endorsement of alcohol products is a particular concern, as they represent direct promotion of new branded alcohol products (including Absolut Tune, Voli Vodka and D'USSE) directly to young consumers. Although there was no direct endorsement of tobacco brands, their presence in music videos is arguably entirely unnecessary and illegal under the 2002 Act 50. In light of consistent evidence, including longitudinal studies, that exposure to tobacco 16, 18, 52 and alcohol 10, 11, 12, 13, 53 promotion and advertising encourages the uptake of smoking and alcohol use in adolescents, our concern is that young people will emulate these smoking and drinking behaviours and therefore that music videos containing alcohol and tobacco content should be restricted. Tobacco and alcohol marketing opportunities should be controlled more tightly in social and user‐generated media, and official music video makers and distributors should be proactive in working towards this goal.

Preventing adolescent exposure to alcohol and tobacco imagery in music videos could be achieved by applying age ratings to the music videos, which would at least provide guidance for parents about the suitability of the content for their child. Videos could also be double‐edited to provide the under‐18s with a ‘cleaner’ version of the video. YouTube's age‐restricted content policy 54 should also ensure that minors are logged out or need to be logged in to view the content, and in the process are age‐verified. However, there are numerous ways in which a child can still watch age‐restricted content, the simplest being to lie about their age. An alternative approach might be to bring pressure on the companies producing the videos to restrict their content, either through consensus or legislation. Other possible preventive measures might include equipping young people with the media literacy skills to promote critical thinking about, and comprehension of, the messages that are being portrayed in these types of media so that they can evaluate them in relation to their own lives, communities and culture.

### Declaration of interests

None.

## Supporting information

Supporting info item
