# Supplementary material for: Adolescents’ exposure to tobacco and alcohol content in YouTube music videos
Source: Addiction. 2015 Jan 28;110(4):703–11. doi: 10.1111/add.12835 (PMC4402034; doi:10.1111/add.12835)
Supplement: Supplementary file 1 — Supporting info item [file add0110-0703-sd1.docx]

**Appendix S1:** Artists and music videos included in the analysis.

| **Artist** | **Video Title** |
| --- | --- |
| Bastiile | Of The Night |
| Calvin Harris | Under Control |
| Jason Derulo | Trumpets |
| Lily Allen | Somewhere Only We Know |
| One Direction | The Story Of My Life |
| Gary Barlow | Let Me Go |
| Eminem And Rihanna | The Monster |
| Ellie Goulding | How Long Will I Love You |
| Avicii | Hey Brother |
| Martin Garrix | Animals |
| Breach Ft Andreya Triana | Everything You Never Had (We Had It All) |
| Little Mix | Move |
| Rebecca Ferguson | I Hope |
| One Republic | Counting Stars |
| Lorde | Royals |
| James Arthur | Recovery |
| All About She | Higher |
| Afro Jack Ft Spree Wilson | The Spark |
| Storm Queen | Look Right Through |
| Awolnation | Sail |
| Katy Perry | Roar |
| Rihanna | What Now |
| Myley Cyrus | Wrecking Ball |
| Avicii | Wake Me Up |
| Elly Goulding | Burn |
| Robbie Williams | Go Gentle |
| Pharrell Williams | Happy |
| Jessie J | Thunder |
| James Blunt | Bonfire Heart |
| Mumford And Sons | I Will Wait |
| Leona Lewis | One More Sleep |
| Katy Perry | Unconditionally |
| Drake | Hold On We're Going Home |
| Ry X | Berlin |
| Olly Murs | Hand On Heart |
| Wilkinson | Afterglow |
| Mcfly | Love Is One The Radio |
| Rudimental Ft Emili Sande | Free |
| Wham | Last Christmas |
| Wizzard | I Wish It Could Be Christmas Every Day |
| Mariah Carey | All I Want For Christmas Is You |
| Pogues Ft Kirsty Maccoll | Fairytale Of New York |
| Chase And Status Ft Jacob Banks | Alive |
| John Newman | Love Me Again |
| Sam Baily | Sky Scraper |
| The Big Reunion | I Wish It Could Be Christmas Every Day |
| Demi Lovat0 | Sky Scraper |
| Kelly Clarkson | Underneath The Tree |
| Ed Sheeran | I See Fire |
| The Killers | Mr Brightside |
| Pitbull Ft K$sha | Timber |
| Ylvis | The Fox |
| Beyonce Ft Jay Z | Drunk In Love |
| Robin Thicke Ft Pharrell Williams | Blurred Lines |
| The Vamps | Can We Dance |
| Jason Derulo | Talk Dirty |
| Rizzle Kicks | Skip To The Good Bit |
| Bastille | Pompeii |
| Little Mix | Little Me |
| One Direction | Best Song Ever |
| Jessy J | It's My Party |
| Sub Focus | Turn Back Time |
| ACDC | Highway To Hell |
| Fuse ODG | Million Pound Girl (Badder Than Bad) |
| Busta Rhymes | Thank You |
| Vance Joy | Riptide |
| Kid Ink Ft Chris Brown | Show Me |
| Elyar Fox | Do It All Over Again |
| Clean Bandit Ft Jess Glynne | Rather Be |
| The Vamps | Wild Heart |
| Neon Jungle | Braveheart |
| Beyonce | Xo |
| Idina Menzel | Let It Go |
| Eminem | Rap God |
| Jason Mraz | I Won't Give Up |
| Reconnected | The Time Of Our Lives |
| Jean Paul | Turn It Up |
| John Newman | Cheating |
| Union J | Beautiful Life |
| Icona Pop | All Night |
| Iggy Azalea | Change Your Life |
| Lady Gaga | Applause |
| M.A.D | Toyboy |
| Chase And Status | Count On Me |
| Klangarussell Ft Will Heard | Sonnentanz (Sun Don't Shine) |
| The Wanted | Show Me The Love (America) |
| Taylor Swift Ft Gary Lightbody | The Last Time |
| Lawson | Juliet |
| Eminem | Survival |
| Avicii | You Make Me |
| Macklemore And Ryan Lewis Ft Mary Lambert | Same Love |
| The Saturdays | Disco Love |
| The Poppy Girls | The Call (No Need To Say Goodbye) |
| The Killers | Shot At The Night |
| Katy B | 5AM |
| James Arthur | You're Nobody Til Somebody Loves You |
| Foxes | Youth |
| Conor Maynard | Ru Crazy |
| Britney Spears | Work Bitch |
| Robin Thicke Ft 2 Chainz | Give It 2U |
| Lily Allen | Hard Out Here |
| JLS | A Billion Lights |
| Showtek/We Are Loved/Wilson | Boo Yah |
| Fatboy Slim/Rivastarr/Beardyman | Eat, Sleep, Rave, Repeat |
| Tinnie Tempah Ft John Martin | Children Of The Sun |
| Passenger | Let Her Go |
| Dizzee Rascal Ft Teddy Sky | Love This Town |
| Keane | Somewhere Only We Know |
| Eminem | Berzerk |
| Boyzone | Love Will Save The Day |
